# Risk factors of proteinuria and potentially protective effect of renin–angiotensin system inhibitors in patients with renal cell carcinoma receiving axitinib

**DOI:** 10.1007/s00280-022-04408-4

**Published:** 2022-03-07

**Authors:** Hiroaki Ikesue, Kenta Yamaoka, Ayako Matsumoto, Masaki Hirabatake, Nobuyuki Muroi, Toshinari Yamasaki, Mutsushi Kawakita, Tohru Hashida

**Affiliations:** 1 Department of Pharmacy, Kobe City Medical Center General Hospital, 2-1-1 Minatojima-Minamimachi, Chuo-ku, Kobe, Hyogo 650-0047 Japan; 2 Department of Urology, Kobe City Medical Center General Hospital, Kobe, Japan

**Keywords:** Axitinib, Renal cell carcinoma, Proteinuria, Risk factor, Renin–angiotensin system inhibitor

## Abstract

**Purpose:**

Patients receiving vascular endothelial growth factor–tyrosine kinase inhibitors are at a risk of developing proteinuria. Renin–angiotensin system (RAS) inhibitors exert renoprotective effects and might reduce proteinuria risk in these patients. We investigated the risk factors for and protective effect of RAS inhibitors against proteinuria in patients with renal cell carcinoma (RCC) receiving axitinib.

**Methods:**

We retrospectively reviewed the medical records of patients with RCC receiving axitinib at Kobe City Medical Center General Hospital between September 2012 and October 2020. Patients with proteinuria ≥ 2+ at baseline were excluded. The patients were categorized into RAS inhibitor user, non-RAS inhibitor user, and non-user groups. The severity of proteinuria was graded based on the Common Terminology Criteria for Adverse Events, version 5.0. A multivariate Cox proportional hazards model was employed to identify the risk factors for developing grade ≥ 2 proteinuria.

**Results:**

Among 42 patients, 28 received antihypertensive drugs at baseline. Among these, 17 and 11 patients were in the RAS inhibitor and non-RAS inhibitor user groups, respectively. Twenty-three patients (54.8%) developed grade ≥ 2 proteinuria. The multivariate analysis revealed that the non-RAS inhibitor user group (*P* = 0.001) and patients with pre-existing grade 1 proteinuria (*P* = 0.022) were significantly associated with the development of grade ≥ 2 proteinuria, whereas the RAS inhibitor user group was not significantly associated with it.

**Conclusion:**

In patients with RCC receiving axitinib, pre-existing proteinuria and non-RAS inhibitor use were significantly associated with grade ≥ 2 proteinuria development. Our preliminary data should be confirmed by further studies.

**Supplementary Information:**

The online version contains supplementary material available at 10.1007/s00280-022-04408-4.

## Introduction

Axitinib, a potent and selective inhibitor of vascular endothelial growth factor (VEGF) receptors 1–3, is recommended as a monotherapy in first- and later-line systemic therapy for metastatic renal cell carcinoma (RCC) [1]. In addition, axitinib and that in combination with immune checkpoint inhibitors are also recommended as first-line therapies for advanced RCC [2, 3]. However, serious adverse drug events (ADEs) are associated with the use of VEGF-targeting agents, including thrombosis, bleeding, hypertension, and proteinuria [4]. Proteinuria is a common ADE and is considered a class effect caused by VEGF-targeting agents [4–6].

The development of proteinuria restricts the dose of VEGF-targeted agents, thereby reducing their efficacy [5, 7–9]. In addition, proteinuria increases the subsequent risk of chronic kidney disease (CKD) [6]. Thus, an understanding of the predictive factors of proteinuria in patients receiving angiogenesis inhibitors will be important for managing this ADE. However, these factors have not been fully elucidated. To the best of our knowledge, there is no information regarding the risk factors of proteinuria in patients with cancer receiving VEGFR–TKIs in real-world clinical settings.

Renin–angiotensin system (RAS) inhibitors are known to exert a renoprotective effect. Several studies have suggested that these agents are useful in reducing the risk of proteinuria in patients receiving VEGF-targeted monoclonal antibodies such as bevacizumab, ramucirumab, and aflibercept [10–12]. Thus, we hypothesized that RAS inhibitors might decrease the risk of development of proteinuria in patients receiving VEGFR–TKIs. Therefore, in this study, we investigated the risk factors for developing proteinuria in patients with RCC receiving axitinib in real-world clinical settings. We also evaluated the protective effect of RAS inhibitors on proteinuria.

## Materials and methods

### Study participants and outcome measures

This retrospective chart review was conducted in accordance with the tenets of the Declaration of Helsinki. The study protocol was approved by the Ethics Committee of the Kobe City Medical Center General Hospital (approval number: zn210301). Patients were eligible if they were ≥ 20 years of age, diagnosed with RCC, and started axitinib monotherapy or combination therapy with immune checkpoint inhibitors at the Department of Urology in Kobe City Medical Center General Hospital between September 1, 2012 and October 31, 2020. A study diagram is shown in Figure S1. The primary objective of this study was to evaluate the risk factors for developing grade ≥ 2 proteinuria in patients with advanced RCC receiving axitinib [10–12]. The secondary objective included determining the risk factors for the exacerbation of proteinuria, and the association between risk factors and cumulative incidence of proteinuria. The exclusion criteria were insufficient data from the urine test (*n* = 16) or an observational period less than 1 month after starting axitinib (*n* = 6). Among the 72 consecutive patients who started axitinib, 50 were enrolled in this study. Subsequently, we investigated the primary objective after excluding patients with baseline proteinuria ≥ 2+ according to urine dipstick testing (*n* = 8).

### Data collection and assessment

Data on pre-existing proteinuria, age, sex, weight, body surface area, Eastern Cooperative Oncology Group performance status, prior nephrectomy, serum creatinine, eGFR, SBP, medications that affect blood pressure (RAS inhibitors, calcium channel blockers, diuretics, and alpha- or beta-blockers), comorbid with diabetes, prior cytokine therapy, and prior targeted therapies were retrospectively collected from electronic medical records. The eGFR was calculated using the formula developed by the Japanese Society of Nephrology [13]. The severity of axitinib-associated proteinuria was graded based on the National Cancer Institute Common Terminology Criteria for Adverse Events (CTCAE), version 5.0. The data cutoff date was December 31, 2020.

### Statistics

Categorical data are presented as number of patients (percentage), and they were compared between groups using Chi-square test or Fisher’s exact test as appropriate. Continuous data are presented as median (interquartile range), and Mann–Whitney *U* test was used to compare the groups. Multivariate Cox proportional hazards models were employed to identify the risk factors for developing grade ≥ 2 proteinuria and exacerbation of proteinuria. We categorized the patients into three groups according to their use of an antihypertensive drug at the start of axitinib as follows: angiotensin receptor blockers or angiotensin-converting enzyme inhibitor users (RAS inhibitor user group), antihypertensive drug users not taking RAS inhibitors (non-RAS inhibitor user group), and patients not using antihypertensive drugs (non-user group) [10, 11]. Variables were examined for multicollinearity (correlation coefficient |*r*|≥ 0.7), because correlations among the variables lead to incorrect results of regression analyses. Covariates were restricted to two variables to avoid overfitting and, based on clinical assessment and previous research, we included pre-existing proteinuria and use of antihypertensive drug at the start of axitinib because of their expected strong associations with the outcome and axitinib treatment. The cumulative incidence of grade ≥ 2 proteinuria was described using the Kaplan–Meier method with the log-rank test. All statistical analyses were performed using JMP 13.2.1 (SAS Institute Inc., Cary NC, USA) and EZR 1.41 (Saitama Medical Center, Jichi Medical University, Saitama, Japan) [14]. Results with two-tailed *P* < 0.05 were considered statistically significant. In the comparison of the cumulative incidence of grade ≥ 2 proteinuria between baseline antihypertensive treatment groups, Bonferroni correction was applied to determine the level of significance between each group and the non-user group (*P* < 0.025).

## Results

### Baseline characteristics of the patients

Between September 2012 and October 2020, 72 consecutive patients with RCC started treatment with axitinib; among them, 50 were enrolled in this study (Figure S1). We divided patients into three groups: RAS inhibitor users (*n* = 23), non-RAS inhibitor users (*n* = 11), and non-users (*n* = 16). The baseline characteristics of these patients are shown in Table 1. There were 39 (78.0%) men and 11 (22.0%) women. Twenty-two patients (44.0%) had proteinuria > 1+ at baseline, according to the urine dipstick test. Axitinib was administered as first-line, second-line, and third-line or later systemic treatment to 9 (18.0%), 12 (24.0%), and 29 (58.0%) patients, respectively. The dose of axitinib was started, in principle, as 10 mg per day, and it was modified during treatment according to the product guidelines. Thirty-four patients (68.0%) concomitantly received one or more antihypertensive agents, consisting of RAS inhibitors (*n* = 23), calcium channel blockers (*n* = 24), or others (*n* = 11).

**Table 1:** Patient characteristics

Characteristics	All patients (*n* = 50)	Non-user (control) (*n* = 16)	RAS inhibitor user (*n* = 23)	Non-RAS inhibitor user (*n* = 11)
Age (years), median (IQR)	67 (62–73)	64 (61–71)	69 (60–74)	68 (65–74)
Male sex, *n* (%)	39 (78.0%)	13 (81.3%)	17 (73.9%)	9 (81.8%)
Weight (kg), median (IQR)	60.0 (51.8–70.0)	59.0 (48.5–64.5)	59.9 (49.0–73.0)	67.0 (59.0–71.0)
Body surface area (m^2^), median (IQR)	1.67 (1.52–1.81)	1.65 (1.49–1.77)	1.62 (1.48–1.79)	1.81 (1.61–1.84)
ECOG PS, *n* (%)				
0	21 (42.0%)	7 (43.8%)	10 (43.5%)	4 (36.4%)
1	24 (48.0%)	9 (56.3%)	10 (43.5%)	5 (45.5%)
2	5 (10.0%)	0 (0%)	3 (13.0%)	2 (18.2%)
Histologic subtype				
Clear cell carcinoma	47 (94.0%)	13 (81.3%)	23 (100%)	11 (100%)
Others	3 (6.0%)	3 (18.7%)	0 (0%)	0 (0%)
IMDC risk group				
Favorable	17 (34.0%)	5 (31.3%)	7 (30.4%)	5 (45.5%)
Intermediate	30 (60.0%)	11 (68.8%)	14 (60.9%)	5 (45.5%)
Poor	2 (4.0%)	0 (0%)	2 (8.7%)	0 (0%)
Unknown	1 (2.0%)	0 (0%)	0 (0%)	1 (9.1%)
Prior nephrectomy, *n* (%)	40 (80.0%)	13 (81.3%)	17 (73.9%)	10 (90.9%)
Pre-existing proteinuria, *n* (%)	22 (44.0%)	4 (25.0%)	14 (60.9%)	4 (36.4%)
eGFR, *n* (%)				
< 45 mL/min/1.73 m^2^	15 (30.0%)	1 (6.3%)	9 (39.1%)	5 (45.5%)
45–59 mL/min/1.73 m^2^	18 (36.0%)	5 (31.3%)	9 (39.1%)	4 (36.4%)
≥ 60 mL/min/1.73 m^2^	17 (34.0%)	10 (62.5%)	5 (21.7%)	2 (18.2%)
SBP (mmHg)	130 (120–135)	130 (121–140)	130 (120–139)	126 (115–130)
Use of antihypertensive agents, *n* (%)	34 (68.0%)	0 (0%)	23 (100%)	11 (100%)
RAS inhibitor	23 (46.0%)	0 (0%)	23 (100%)	0 (0%)
Calcium channel blocker	24 (48.0%)	0 (0%)	14 (60.9%)	10 (90.9%)
Other drugs	11 (22.0%)	0 (0%)	9 (39.1%)	2 (18.2%)
Comorbid with diabetes, *n* (%)	13 (26.0%)	2 (14.3%)	8 (36.4%)	3 (27.3%)
Line of therapy				
1st	9 (18.0%)	2 (12.5%)	4 (17.4%)	3 (27.3%)
2nd	12 (24.0%)	5 (31.3%)	6 (26.1%)	1 (9.1%)
3rd	13 (26.0%)	5 (31.3%)	6 (26.1%)	2 (18.2%)
4th or later	16 (32.0%)	4 (25.0%)	7 (30.4%)	5 (45.5%)
Prior cytokine therapy, *n* (%)	23 (46.0%)	8 (50.0%)	10 (43.5%)	5 (45.5%)
Prior targeted therapy, *n* (%)				
Sunitinib	20 (40.0%)	6 (37.5%)	8 (34.8%)	6 (54.6%)
Everolimus	10 (20.0%)	4 (25.0%)	5 (21.7%)	1 (9.1%)
Sorafenib	10 (20.0%)	2 (12.5%)	6 (26.1%)	2 (18.2%)
Pazopanib	10 (20.0%)	3 (18.7%)	5 (21.7%)	2 (18.2%)
Temsirolimus	2 (4.0%)	1 (6.3%)	0 (0%)	1 (9.1%)
Prior ICI, *n* (%)	4 (8.0%)	1 (6.3%)	2 (8.7%)	1 (9.1%)
Axitinib monotherapy	43 (86.0%)	15 (93.8%)	19 (82.6%)	9 (81.8%)
Axitinib and ICI combination therapy	7 (14.0%)	1 (6.3%)	4 (17.4%)	2 (18.2%)
Duration of axitinib treatment, months (IQR)	7.3 (2.7–13.9)	7.8 (1.9–14.4)	6.5 (2.5–16.2)	8.6 (3.2–13.1)

*IQR* interquartile range, *ECOG* *PS* Eastern Cooperative Oncology Group performance status, *IMDC* International Metastatic Renal Cell Carcinoma Database Consortium, *eGFR* estimated glomerular filtration rate, *SBP* systolic blood pressure, *RAS* renin–angiotensin system, *ICI* immune checkpoint inhibitor

The baseline characteristics of 42 patients with baseline proteinuria < 2+ , as determined using the urine dipstick test, are shown in Supplementary Table S1. There were 34 (81.0%) men and 8 (19.0%) women. Fourteen patients (33.3%) had proteinuria 1+ at baseline, according to the urine dipstick test. Twenty-eight patients (66.7%) concomitantly received one or more antihypertensive agents. The proportion of patients with pre-existing proteinuria at baseline (47.1 vs. 36.4%) was relatively higher but not statistically different between the RAS inhibitor user and non-RAS inhibitor user groups.

### Incidence and risk factors of proteinuria

Among the 50 patients, 38 (76.0%) presented exacerbated grade of proteinuria (Supplementary Table S2). According to the analysis using the multivariate Cox proportional hazards model, the non-RAS inhibitor user group (hazard ratio [HR] 5.21; 95% confidence interval [CI], 1.99–14.17; *P* = 0.001) was significantly associated with an exacerbated grade of proteinuria. In contrast, the RAS inhibitor user group showed no significant association with it (HR 1.32; 95% CI 0.56–3.26; *P* = 0.531) (Supplementary Table S3). The Kaplan–Meier curve for the cumulative incidence of an exacerbated grade of proteinuria after starting axitinib treatment is shown in Supplementary Figure S2.

Among the 42 patients with baseline proteinuria < 2+ detected using the urine dipstick test, grade 1, 2, and 3 proteinuria was observed in 14 (21.4%), 22 (54.8%), and 1 (4.8%) patient(s), respectively, during axitinib treatment. To investigate the risk factors for developing grade ≥ 2 proteinuria, multivariate Cox proportional hazards models were employed. The non-RAS inhibitor user group (HR 7.52; 95% CI 2.29–29.19; *P* = 0.001) and patients with pre-existing proteinuria (HR 2.98; 95% CI 1.17–7.60; *P* = 0.022) were significantly associated with the development of grade ≥ 2 proteinuria (Table S4), in contrast to the RAS inhibitor user group (HR 1.77; 95% CI 0.54–6.88; *P* = 0.352).

The incidence of developing each grade of proteinuria in the non-user, RAS inhibitor user, and non-RAS inhibitor user groups as well as in patients with and without pre-existing proteinuria at baseline are shown in Table S5. The incidence of developing grade ≥ 2 proteinuria was significantly different among the non-user, RAS inhibitor user, and non-RAS inhibitor user groups [28.6% (4/14), 58.8% (10/17), and 81.8% (9/11), respectively; *P* = 0.030]. The incidence of developing grade ≥ 2 proteinuria was also significantly different between patients with and without pre-existing proteinuria [78.6% (11/14) vs. 42.9% (12/28), respectively; *P* = 0.048].

**Fig. 1:**
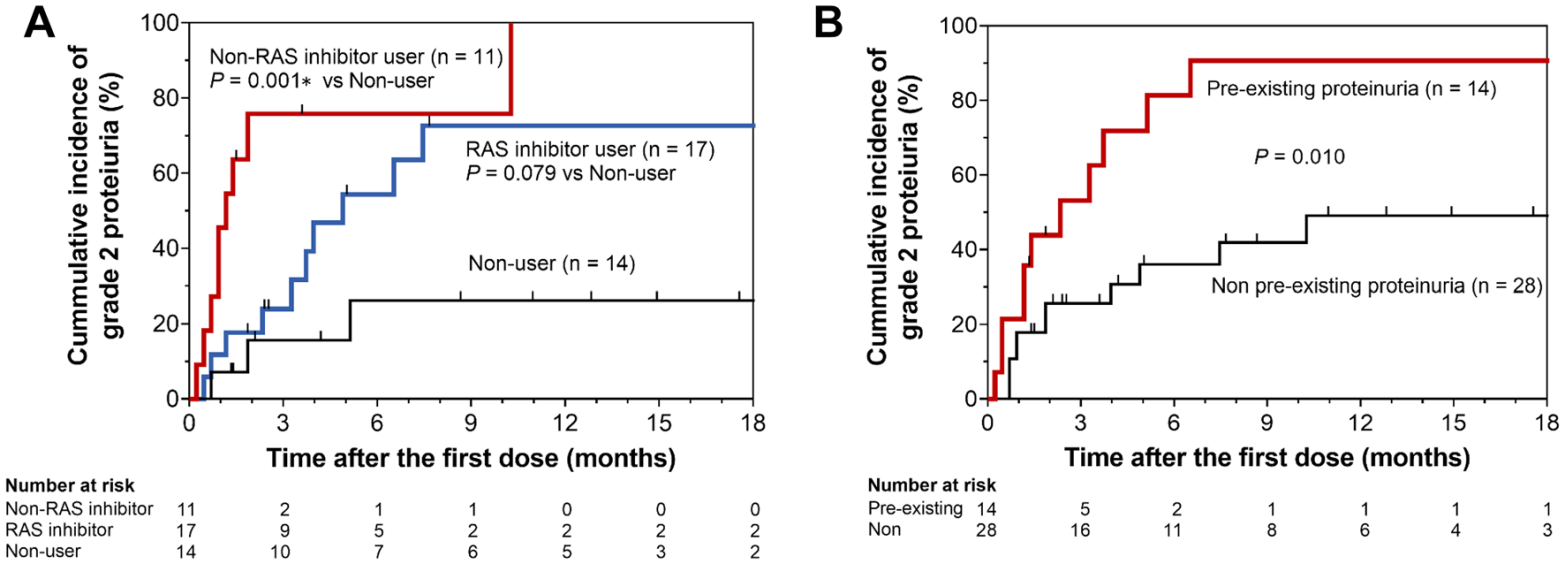
Kaplan–Meier curves for the cumulative incidence of grade 2 proteinuria in patients with renal cell carcinoma receiving axitinib. Cumulative incidence of grade 2 proteinuria was compared (**A**) among the RAS inhibitor user, non-RAS inhibitor user, and non-user groups and (**B**) between patients with and without pre-existing proteinuria. *Statistically significant after adjustment using Bonferroni correction (*P* < 0.025 for log-rank test). RAS, renin–angiotensin system

The Kaplan–Meier curve for the cumulative incidence of proteinuria after starting axitinib is shown in Fig. 1. The cumulative incidence of developing grade ≥ 2 proteinuria (Fig. 1A) was significantly higher in the non-RAS inhibitor user group than in the non-user group (*P* = 0.001). In contrast, there was no such significant difference between the RAS inhibitor user and non-user groups (*P* = 0.079). Finally, the cumulative incidence of developing grade ≥ 2 proteinuria (Fig. 1B) was significantly higher in patients with pre-existing proteinuria at baseline than in those without (*P* = 0.010).

## Discussion

The multivariate Cox proportional hazards model used in this study showed that the risk of developing grade ≥ 2 proteinuria was significantly higher in patients with proteinuria at baseline and in the non-RAS inhibitor group. In contrast, it was not significantly higher in the RAS inhibitor user group. To the best of our knowledge, this is the first study on the risk factors of proteinuria in patients with cancer receiving VEGFR–TKIs in a real-world clinical setting.

In a pooled analysis of data from two phase III randomized controlled trials in patients with metastatic RCC receiving pazopanib or sunitinib (*n* = 1392), it was found that Asian ethnicity, diabetes, hypertension, pre-existing proteinuria, and prior nephrectomy are significant risk factors for proteinuria [9]. Owing to the small number of patients in this study, we did not employ baseline lower kidney function as one of potential risk factors for proteinuria. In our study, pre-existing proteinuria was a significant risk factor for developing grade ≥ 2 proteinuria.

A novel finding of our study was that patients treated with the concomitant use of RAS inhibitors at baseline showed a significantly reduced risk of proteinuria among those receiving oral VEGFR–TKIs. However, the non-RAS inhibitor user group did not show a significant reduction in the risk of developing grade ≥ 2 proteinuria compared with that in the non-user group. It has been reported that the concomitant use of RAS inhibitors at baseline significantly reduces the risk of proteinuria in patients receiving intravenous monoclonal antibodies targeting the human VEGF pathway, such as bevacizumab, ramucirumab, and aflibercept [10–12]. In controlled trials of patients with CKD, RAS inhibitors reduced proteinuria by approximately 35–40% and were thus more effective than other antihypertensive drugs [15, 16]. With regard to the mechanisms of renal protection mediated by RAS inhibitors, both renal hemodynamic and non-hemodynamic effects might be involved [17]. Selecting RAS inhibitors to control this condition in patients receiving VEGFR–TKIs could be an important option.

There were some limitations to our study. First, this was a single-centered, small, retrospective study. Although a phase II trial of axitinib monotherapy for patients with metastatic RCC (*n* = 64) showed that baseline urine protein level and lower eGFR are significantly associated with proteinuria [8], we did not employ these factors as potential risk factors for proteinuria owing to the small number of patients in this study. Second, the study subjects consisted of a heterogeneous population on first to later lines of TKI treatment. The proportion of patients with pre-existing proteinuria was relatively higher in the RAS inhibitor user group than in the other groups. It is noteworthy that the development of grade ≥ 2 proteinuria was decreased in the RAS inhibitor user group, despite an imbalance in the proportion of patients with pre-existing proteinuria at baseline. Third, although recent studies have reported that evaluating the urine protein/creatinine ratio (UPCR) might be appropriate for assessing proteinuria in patients with advanced RCC receiving VEGFR–TKIs [18], we did not use the UPCR, because it has not been utilized in the CTCAE criteria and the protocols of randomized controlled trials. Finally, we did not precisely quantitate 24 h urine protein based on the retrospective observational study design. Our preliminary findings should thus be confirmed in further studies.

In conclusion, we investigated the risk of developing grade ≥ 2 proteinuria in patients with RCC receiving axitinib either as monotherapy or in combination with immune checkpoint inhibitors. The results of our study suggest that patients with pre-existing proteinuria at baseline and non-RAS inhibitor user groups are significantly associated with developing grade ≥ 2 proteinuria. Our preliminary findings should be confirmed by prospective studies with a higher number of patients.

## Supplementary Information

Below is the link to the electronic supplementary material.

Supplementary file1 (DOCX 51 KB)

## Data Availability

All data generated or analyzed during this study are included in this published article and its supplementary information files.

## References

[1] Rini BI, Escudier B, Tomczak P, Kaprin A, Szczylik C, Hutson TE, Michaelson MD, Gorbunova VA, Gore ME, Rusakov IG, Negrier S, Ou YC, Castellano D, Lim HY, Uemura H, Tarazi J, Cella D, Chen C, Rosbrook B, Kim S, Motzer RJ (2011). Comparative effectiveness of axitinib versus sorafenib in advanced renal cell carcinoma (AXIS): a randomised phase 3 trial. Lancet.

[2] Rini BI, Plimack ER, Stus V, Gafanov R, Hawkins R, Nosov D, Pouliot F, Alekseev B, Soulieres D, Melichar B, Vynnychenko I, Kryzhanivska A, Bondarenko I, Azevedo SJ, Borchiellini D, Szczylik C, Markus M, McDermott RS, Bedke J, Tartas S, Chang YH, Tamada S, Shou Q, Perini RF, Chen M, Atkins MB, Powles T, Investigators K (2019). Pembrolizumab plus axitinib versus sunitinib for advanced renal-cell carcinoma. N Engl J Med.

[3] Motzer RJ, Penkov K, Haanen J, Rini B, Albiges L, Campbell MT, Venugopal B, Kollmannsberger C, Negrier S, Uemura M, Lee JL, Vasiliev A, Miller WH, Gurney H, Schmidinger M, Larkin J, Atkins MB, Bedke J, Alekseev B, Wang J, Mariani M, Robbins PB, Chudnovsky A, Fowst C, Hariharan S, Huang B, di Pietro A, Choueiri TK (2019). Avelumab plus axitinib versus sunitinib for advanced renal-cell carcinoma. N Engl J Med.

[4] Zhu X, Wu S, Dahut WL, Parikh CR (2007). Risks of proteinuria and hypertension with bevacizumab, an antibody against vascular endothelial growth factor: systematic review and meta-analysis. Am J Kidney Dis.

[5] Zhang W, Feng LJ, Teng F, Li YH, Zhang X, Ran YG (2020). Incidence and risk of proteinuria associated with newly approved vascular endothelial growth factor receptor tyrosine kinase inhibitors in cancer patients: an up-to-date meta-analysis of randomized controlled trials. Expert Rev Clin Pharmacol.

[6] Izzedine H, Massard C, Spano JP, Goldwasser F, Khayat D, Soria JC (2010). VEGF signalling inhibition-induced proteinuria: mechanisms, significance and management. Eur J Cancer.

[7] Nozawa M, Sugimoto K, Ohzeki T, Minami T, Shimizu N, Adomi S, Saito Y, Nose K, Yoshimura K, Uemura H (2016). Axitinib-induced proteinuria and efficacy in patients with metastatic renal cell carcinoma. Int J Clin Oncol.

[8] Tomita Y, Uemura H, Fujimoto H, Kanayama HO, Shinohara N, Nakazawa H, Imai K, Umeyama Y, Ozono S, Naito S, Akaza H, Japan Axitinib Phase II Study Group (2011). Key predictive factors of axitinib (AG-013736)-induced proteinuria and efficacy: a phase II study in Japanese patients with cytokine-refractory metastatic renal cell carcinoma. Eur J Cancer.

[9] Sorich MJ, Rowland A, Kichenadasse G, Woodman RJ, Mangoni AA (2016). Risk factors of proteinuria in renal cell carcinoma patients treated with VEGF inhibitors: a secondary analysis of pooled clinical trial data. Br J Cancer.

[10] Nihei S, Sato J, Harada T, Kuyama S, Suzuki T, Waga N, Saito Y, Kisara S, Yokota A, Okada K, Tsuchiya M, Terui K, Tadokoro Y, Chiba T, Kudo K, Oizumi S, Inoue A, Morikawa N (2018). Antiproteinuric effects of renin–angiotensin inhibitors in lung cancer patients receiving bevacizumab. Cancer Chemother Pharmacol.

[11] Hirai T, Shuji Y, Takiyama M, Hanada K, Itoh T (2019). Renin–angiotensin system inhibitors for countering proteinuria induced by angiogenesis inhibitors: a retrospective observational analysis. Cancer Chemother Pharmacol.

[12] Kanbayashi Y, Ishikawa T, Tabuchi Y, Sakaguchi K, Ouchi Y, Otsuji E, Takayama K, Taguchi T (2020). Predictive factors for the development of proteinuria in cancer patients treated with bevacizumab, ramucirumab, and aflibercept: a single-institution retrospective analysis. Sci Rep.

[13] Matsuo S, Imai E, Horio M, Yasuda Y, Tomita K, Nitta K, Yamagata K, Tomino Y, Yokoyama H, Hishida A, Collaborators developing the Japanese equation for estimated GFR, (2009). Revised equations for estimated GFR from serum creatinine in Japan. Am J Kidney Dis.

[14] Kanda Y (2013). Investigation of the freely available easy-to-use software ‘EZR’ for medical statistics. Bone Marrow Transplant.

[15] Maki DD, Ma JZ, Louis TA, Kasiske BL (1995). Long-term effects of antihypertensive agents on proteinuria and renal function. Arch Intern Med.

[16] Gansevoort RT, Sluiter WJ, Hemmelder MH, de Zeeuw D, de Jong PE (1995). Antiproteinuric effect of blood-pressure-lowering agents: a meta-analysis of comparative trials. Nephrol Dial Transplant.

[17] Lewis EJ, Hunsicker LG, Clarke WR, Berl T, Pohl MA, Lewis JB, Ritz E, Atkins RC, Rohde R, Raz I, Collaborative Study G (2001). Renoprotective effect of the angiotensin-receptor antagonist irbesartan in patients with nephropathy due to type 2 diabetes. N Engl J Med.

[18] Evans TRJ, Kudo M, Finn RS, Han KH, Cheng AL, Ikeda M, Kraljevic S, Ren M, Dutcus CE, Piscaglia F, Sung MW (2019). Urine protein:creatinine ratio vs 24-hour urine protein for proteinuria management: analysis from the phase 3 REFLECT study of lenvatinib vs sorafenib in hepatocellular carcinoma. Br J Cancer.

