# Supplementary material for: Risk factors of proteinuria and potentially protective effect of renin–angiotensin system inhibitors in patients with renal cell carcinoma receiving axitinib
Source: Cancer Chemother Pharmacol. 2022 Mar 7;89(6):833–8. doi: 10.1007/s00280-022-04408-4 (PMC9135790; doi:10.1007/s00280-022-04408-4)
Supplement: Supplementary file 1 — Supplementary file1 (DOCX 51 KB) [file 280_2022_4408_MOESM1_ESM.docx]

Supplementary Materials

# Supplementary Figures

Patients that received axitinib (n = 72)

Excluded

· Insufficient urine test data (n = 16)

· Follow-up period < 1 month (n = 6)

Patients (n = 50)

Secondary objectives

· Risk factors for exacerbation of proteinuria

Excluded

· Baseline proteinuria ≥ 2+ according to

urine dipstick testing (n = 8)

Patients (n = 42)

Primary objective

· Risk factors for developing grade ≥ 2 proteinuria

Secondary objective

· Association between risk factors and cumulative

incidences of proteinuria

Supplementary Figure S1. **Study diagram.**

Supplementary Figure S2. **Kaplan–Meier curve for cumulative incidence of exacerbated grade of proteinuria.**

Cumulative incidences of exacerbated grade of proteinuria in 50 patients were compared among the RAS inhibitor user, non-RAS inhibitor user, and non-user groups. *, Statistically significant after adjustment using the Bonferroni correction (*P* < 0.025 for log-rank test). RAS, renin-angiotensin system.

Supplementary Table S1**.** Patient characteristics in 42 patients with baseline proteinuria <2+ detected by urine dipstick test

| Characteristics | All patients  (n = 42) | Non-user  (control)  (n = 14) | RAS inhibitor  user  (n = 17) | Non-RAS inhibitor user  (n = 11) |
| --- | --- | --- | --- | --- |
| Age (years), median (IQR) | 66 (61–74) | 64 (60–69) | 67 (59–81) | 68 (65–74) |
| Male sex, n (%) | 34 (81.0%) | 11 (78.6%) | 14 (82.4%) | 9 (81.8%) |
| Weight (kg), median (IQR) | 60.0 (51.8–71.0) | 59.0 (47.5–65.8) | 59.9 (49.5–75.0) | 67.0 (59.0–71.0) |
| Body surface area (m^2^), median (IQR) | 1.67 (1.52–1.83) | 1.65 (1.48–1.77) | 1.62 (1.50–1.90) | 1.81 (1.61–1.84) |
| ECOG PS, n (%) |  |  |  |  |
| 0 | 18 (42.9%) | 6 (42.9%) | 8 (47.1%) | 4 (36.4%) |
| 1 | 19 (45.2%) | 8 (57.1%) | 6 (35.3%) | 5 (45.5%) |
| 2 | 5 (11.9%) | 0 (0%) | 3 (17.6%) | 2 (18.2%) |
| Histologic subtype  Clear cell carcinoma  Others | 40 (95.2%)  2 (4.8%) | 12 (85.7%)  2 (14.3%) | 17 (100%)  0 (0%) | 11 (100%)  0 (0%) |
| IMDC risk group  Favorable  Intermediate  Poor  Unknown | 13 (31.0%)  26 (61.9%)  2 (4.8%)  1 (2.4%) | 4 (28.6%)  10 (71.4%)  0 (0%)  0 (0%) | 4 (23.5%)  11 (64.7%)  2 (11.8%)  0 (0%) | 5 (45.5%)  5 (45.5%)  0 (0%)  1 (9.1%) |
| Prior nephrectomy, n (%) | 35 (83.3%) | 12 (85.7%) | 13 (76.5%) | 10 (90.9%) |
| Pre-existing proteinuria, n (%) | 14 (33.3%) | 2 (14.3%) | 8 (47.1%) | 4 (36.4%) |
| eGFR, n (%) |  |  |  |  |
| <45 mL/min/1.73 m^2^ | 11 (26.2%) | 0 (0%) | 6 (35.3%) | 5 (45.5%) |
| 45–59 mL/min/1.73 m^2^ | 15 (35.7%) | 5 (35.7%) | 6 (35.3%) | 4 (36.4%) |
| ≥60 mL/min/1.73 m^2^ | 16 (38.1%) | 9 (64.3%) | 5 (29.4%) | 2 (18.2%) |
| SBP (mmHg) | 128 (120–135) | 129 (120–138) | 130 (120–140) | 126 (115–130) |
| Use of antihypertensive agents, n (%) | 28 (66.7%) | 0 (0%) | 17 (100%) | 11 (100%) |
| RAS inhibitor | 17 (40.5%) | 0 (0%) | 17 (100%) | 0 (0%) |
| Calcium channel blocker | 20 (47.6%) | 0 (0%) | 10 (58.8%) | 10 (90.9%) |
| Other drugs | 6 (14.3%) | 0 (0%) | 4 (23.5%) | 2 (18.2%) |
| Comorbid with diabetes, n (%) | 10 (23.8%) | 2 (14.3%) | 5 (29.4%) | 3 (27.3%) |
| Line of therapy |  |  |  |  |
| 1st | 7 (16.7%) | 1 (7.1%) | 3 (17.6%) | 3 (27.3%) |
| 2nd | 11 (26.2%) | 5 (35.7%) | 5 (29.4%) | 1 (9.1%) |
| 3rd | 11 (26.2%) | 4 (28.6%) | 5 (29.4%) | 2 (18.2%) |
| 4th or later | 13 (31.0%) | 4 (28.6%) | 4 (23.5%) | 5 (45.5%) |
| Prior cytokine therapy, n (%) | 20 (47.6%) | 8 (57.1%) | 7 (41.2%) | 5 (45.5%) |
| Prior targeted therapy, n (%) |  |  |  |  |
| Sunitinib | 18 (47.6%) | 6 (42.9%) | 6 (35.3%) | 6 (54.6%) |
| Everolimus | 7 (16.7%) | 3 (21.4%) | 3 (17.6%) | 1 (9.1%) |
| Sorafenib | 7 (16.7%) | 2 (14.3%) | 3 (17.6%) | 2 (18.2%) |
| Pazopanib | 6 (14.3%) | 2 (14.3%) | 2 (11.8%) | 2 (18.2%) |
| Temsirolimus | 2 (4.8%) | 1 (7.1%) | 0 (0%) | 1 (9.1%) |
| Prior ICI, n (%) | 2 (4.8%) | 1 (7.1%) | 0 (0%) | 1 (9.1%) |
| Axitinib monotherapy  Axitinib and ICI combination therapy | 36 (85.7%)  6 (14.3%) | 13 (92.9%)  1 (7.1%) | 14 (82.4%)  3 (17.6%) | 9 (81.8%)  2 (18.2%) |
| Duration of axitinib treatment, months (IQR) | 8.2 (3.1–13.9) | 7.8 (2.0–15.6) | 7.7 (3.7–16.1) | 8.6 (3.2–13.1) |

IQR, interquartile range; ECOG PS, Eastern Cooperative Oncology Group performance status; IMDC, International Metastatic Renal Cell Carcinoma Database Consortium; eGFR, estimated glomerular filtration rate; SBP, systolic blood pressure; RAS, renin-angiotensin system; ICI, immune checkpoint inhibitor

**Supplementary Table S2.** Relationship between grades of proteinuria at baseline and after starting axitinib treatment

|  | Proteinuria after starting axitinib treatment | | | |
| --- | --- | --- | --- | --- |
|  | Grade 0 | Grade 1 | Grade 2 | Grade 3 |
| Proteinuria at baseline |  |  |  |  |
| Grade 0 (n = 28) | 5 | 11 | 12 | 0 |
| Grade 1 (n = 14) | 0 | 3 | 10 | 1 |
| Grade 2 (n = 8) | 0 | 0 | 4 | 4 |

Shaded cells show the number of patients with exacerbated grade of proteinuria (38/50, 76.0%) after starting axitinib treatment.

# Supplementary Table S3. Multivariate Cox proportional hazards model for exacerbated grade of proteinuria in patients receiving axitinib (n = 50).

| Variables | Multivariate analysis | |
| --- | --- | --- |
|  | HR (95% CI) | *P*-value |
| Antihypertensive agents |  |  |
| Non-user (control) | 1.00 | – |
| RAS inhibitor user | 1.32 (0.56–3.26) | 0.531 |
| Non-RAS inhibitor user | 5.21 (1.99–14.17) | 0.001 |
| Pre-existing proteinuria | 0.74 (0.35–1.55) | 0.430 |

HR, hazard ratio; CI, confidence interval; RAS, renin-angiotensin system.

# Table S4. Multivariate Cox proportional hazards model for development of grade 2 proteinuria in patients receiving axitinib (n = 42)

| Variables | Multivariate analysis | |
| --- | --- | --- |
|  | HR (95% CI) | *P*-value |
| Antihypertensive agents |  |  |
| Non-user (control) | 1.00 | – |
| RAS inhibitor user | 1.77 (0.54–6.88) | 0.352 |
| Non-RAS inhibitor user | 7.52 (2.29–29.19) | 0.001 |
| Pre-existing proteinuria | 2.98 (1.17–7.60) | 0.022 |

HR, hazard ratio; CI, confidence interval; RAS, renin-angiotensin system.

**Table S5.** Incidences of proteinuria in patients receiving axitinib

| Variables |  | Grade | | | |
| --- | --- | --- | --- | --- | --- |
|  | n | 0 | 1 | 2 | 3 |
| Use of antihypertensive agents |  |  |  |  |  |
| Non-user (control) | 14 | 4 (28.6%) | 6 (42.9%) | 4 (28.6%) | 0 (0%) |
| RAS inhibitor user | 17 | 1 (5.9%) | 6 (35.3%) | 9 (52.9%) | 1 (5.9%) |
| Non-RAS inhibitor user | 11 | 0 (0%) | 2 (18.2%) | 9 (81.8%) | 0 (0%) |
| Pre-existing proteinuria |  |  |  |  |  |
| Yes | 14 | 0 (0%) | 3 (21.4%) | 10 (71.4%) | 1 (7.1%) |
| No | 28 | 5 (17.9%) | 11 (39.3%) | 12 (42.9%) | 0 (0%) |

RAS, renin-angiotensin system.
